# Supercritical CO_2_ Extraction of Extracted Oil from *Pistacia lentiscus* L.: Mathematical Modeling, Economic Evaluation and Scale-Up

**DOI:** 10.3390/molecules25010199

**Published:** 2020-01-03

**Authors:** Abdelkarim Aydi, André Wüst Zibetti, Abdulaal Z. Al-Khazaal, Aboulbaba ELADEB, Manef ADBERRABA, Danielle BARTH

**Affiliations:** 1 Laboratory Materials, Molecules and Applications, Preparatory Institute for Scientific and Technical Studies, 2070 Marsa, Tunisia; eladebboulbaba@gmail.com (A.E.); manef.abderrabba@ipest.RNU.tn (M.A.); 2 Laboratorio de Controle de Processos, Departments of Chemical Engineering and Food Engineering, Universidade Federal de Santa Catarina (UFSC), P.O. Box 476, Florianópolis 88010-970, Brazil; azibetti@gmail.com; 3 Department of Chemical and Materials Engineering, Faculty of Engineering, Northern Border University, Arar P.O. Box 1321, Saudi Arabia; abdulaal.alkhazaal@gmail.com; 4 Laboratoire Réactions et Génie des Procédés, Université de Lorraine, CNRS, LRGP F-5400 Nancy, France; danielle.barth@univ-lorraine.fr

**Keywords:** supercritical carbon dioxide (SC-CO_2_) extraction, *Pistacia lentiscus* L., response surface methodology, diffusion coefficient, mass transfer parameter, economic study

## Abstract

In this study, the extracted oil of *Pistacia lentiscus* L. the Tunis region was extracted using supercritical carbon dioxide (SC-CO_2_) extraction containing different major components in the oil such as α-pinene (32%) and terpinene-4-ol (13%). The investigation of the effect of different variables on the extraction yield with 5% level of confidence interval showed that the CO_2_ pressure was the main significant variable to influence the oil yield. In order to better understand the phenomena, three parameters were considered to adjust all parameters of broken and intact cell (BIC) model: grinding efficiency (G), the internal mass transfer parameter ($k_{S}a_{0}$), and the external mass transfer parameter ($k_{f}a_{0}$), which were estimated by experimental extraction curves to calculate the diffusion coefficient. From an economic point of view, we found out that the high cost of production of the extracted oil was due to the low mass of extracted oil obtained from this type of plant.

## 1. Introduction

Plant species with medicinal properties are becoming crucially essential in research due to their utilities in many areas such as traditional medicine, food, cosmetics and pharmaceuticals [1]. In Tunisia, a species of plant called *Pistacia lentiscus* L., commonly known as “Dharw” in the Maghreb and “Elustaka” in the Middle East, has medicinal properties in its oil extract. The extracted oil has numerous medicianal properties, for instance anti-atherogenic [2], anti-inflammatory [3,4,5], anti-oxidant [6,7,8,9,10], antimicrobial [9,11,12,13], hypotensive [14,15], anticancer [5,16], anti-arthritis [17], wound treatment, and anti-asthmatic and anthelmintic activities [18,19,20].

The essential oil can be extracted from specific plants by several extraction techniques: hydro-distillation [21], Soxhlet extraction [22] and supercritical carbon dioxide (SC-CO_2_) extraction [23,24,25]. The major objective of the extraction process is to provide a more concentrated form of the desired material. Although the cost never compromises the quality, it can be a decisive factor in choosing an adequate process. However, the extraction effectiveness and the safety process must be priorities. In fact, as the limits of solvent residues are increasingly subjected to review, the supercritical solvent used to extract the oil in supercritical fluid extraction (SFE) can replace toxic solvents. To obtain the desired product which meets the needs of consumers, this can be a key element in defining the extract quality.

Modeling of SC-CO_2_ extraction from natural matter is a very important tool and it represents a challenge in the research field. Many models have been developed and are currently used in supercritical fluid extraction [26,27,28,29,30]. The most widely used model was developed by Sovová [28,29]. This model is known as the broken and intact cell (BIC) model. It assumes diffusive and convective transport phenomena during the extraction that occurs in three periods. Also, he developed an analytical solution to estimate the extraction parameters by comparing the results of the extraction curves calculated by the model with the experimental data. Stástova et al. [30] simplified the model developed by Sovová [29] to describe the buckthorn extraction curves to evaluate grinding efficiency, mass transfer coefficients, and flow asymmetry.

To the best of our knowledge, the bulk of the prior research involving the extraction of essential oils of *Pistacia lentiscus* are focused on hydro-distillation extraction. Few studies have reported the supercritical extraction process for the plant of *Pistacia lentiscus* (PL). Congiu et al. [31] isolated the essential oil from the leaves and the berries of *Pistacia lentiscus* collected in the region of Sardinia (Costa Rey and Capoterra) using a supercritical CO_2_ extraction technique coupled with the fractional separation technique (SFE). Thus, the authors separated the essential oil from cuticular waxes. The obtained yields of the extracted oil from the leaves and the berries were 0.45% and 0.20%, respectively, with the presence of major compounds such as β-caryophyllene, germacrene, β-myrcene, and α-pinene.

It is well-known that the quantity of the extract yield from supercritical CO_2_ extraction is affected by several operating parameters such as the CO_2_ pressure, CO_2_ mass flow rate, time of extraction, and average particle size. In order to optimize the extract yield of the oil produced from the leaves, an efficient way might be to systematically create models around the key ingredient levels of the product via some type of response surface experimental design [32]. Response surface methodology (RSM) is a collection of mathematical and statistical techniques that make a full description of the effect of independent variables near the optimum conditions [21,33,34]. Several classes of treatment structures can be used as RSM experiments [35].

The main objective of our study is to use the response surface methodology (RSM) to study the effect of three operating conditions (pressure, average particle size, and CO_2_ flow rate) and their interaction on the extract yield. We also aim to study the influence of operating parameters on mass transfer by evaluating a process applying the BIC model proposed by Sovová [29] in 1994 which was simplified later by Stástova et al. [30] on the essential oil extraction curves that are acquired from *Pistacia lentiscus* leaves growing in the northern parts of Tunisia. In the end, we obtained an economic evaluation in the scale-up process for the CO_2_ extract of these plant leaves.

## 2. Materials and Methods

### 2.1. Materials

The *Pistacia lentiscus* (PL) plant leaves were obtained from a local market in Tunis (Tunisia). The leaves were air-dried under a controlled temperature of 37 °C for 48 h, then stored in vacuum-sealed plastic bags under refrigeration prior to extraction. Immediately prior to supercritical fluid extraction, only the leaves of the samples were used, and they were rounded in a blender to get particle powder with diameters 220 µm and 650 µm. The chemicals used were absolute ethanol (Carlo Erba, Val de Reuil, France), ultrapure water and carbon dioxide (Messer Group, Nancy, France, 99.95%).

### 2.2. The Procedure of Supercritical Fluid Extraction

**Figure 1:**
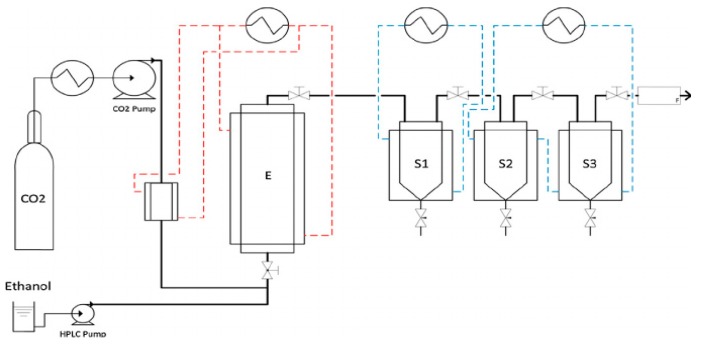
Fluid extraction and fractionation unit schematic drawing. E: Extractor; S1, S2, S3: Separators [34].

The main purpose of using supercritical fluid extraction (SFE) was to obtain the extracted oils. The SFE apparatus is shown in Figure 1.

The extract was collected as a function of time during the process through valves located at the base of the separators. The samples were weighed after 30 min of collection to avoid measuring CO_2_ remaining in the bottle.

The temperature of extraction was maintained constant in all the experiments (40 °C) to prevent the heat degradation of thermolabile components in the extracted oil. Table 1 shows the experimental conditions of the SFE unit where *P* is CO_2_ pressure, $Q_{{\mathrm{CO}}_{2}}$ is CO_2_ flow rate, *d_P_* is the average particle size of the leave, $\rho_{{\mathrm{CO}}_{2}}$ is CO_2_ density, and $\mu_{{\mathrm{CO}}_{2}}$ is CO_2_ velocity. Observing Table 1, we noted that the conditions of extraction used during these experiments show that the CO_2_ flow was manually controlled, and the estimated variance of the experiments is between 2.5% to 5% of the average flow.

Referring to a previous publication [29] and in order to ensure the solubility of major compounds [36,37,38,39], the collection of extracted oils and waxes was conducted using separators. The first separator was maintained at a low temperature (−5 °C) with the same extraction pressure as the experiment to precipitate waxes while the second separator was maintained at 30 °C and 40 bar for oil extract collection.

The bulk density of milled *Pistacia* leaves was about 291 kg m^−3^, and the void fraction of the bed was equal to 0.53. Glass beads were placed on the bottom of the extractor, the powder of *Pistacia* leaves (23 ± 0.05 g) was placed above them and another layer of glass bead was put on the top. In addition, two filters (frits <15 µm) were used in both the inlet and the outlet of the extracting vessel.

### 2.3. Analysis

#### Gas Chromatography-Flame Ionization Detector/Mass Spectrometry (GC-FID/MS)

GC-FID analysis was carried out with a Shimadzu GC2010 Plus (Nancy, France), equipped with an HP-5 capillary column (Shimadzu, Nancy, France, with dimension: 30 m × 0.25 mm, film thickness 0.25 µm). The injector and the detector were set at 250 and 300 °C. The temperature column was programmed at 50 °C for 1 min then gradually increased to 270 °C at 3 °C/min. Next, it was held for 5 min then increased to 300 °C at 20 °C/min and subsequently held for 5 min. The split ratio was 5:1 whereas the split flow was equal to 10 mL/min. Nitrogen was used as a carrier gas with a constant pressure of 100 kPa. The GC was also equipped with an Auto-Injector (Shimadzu, Nancy, France, AOC-20i) and the injected volume was equal to 1 µL.

For GC-FID-MS analysis, a Shimadzu GCMS-GC2010-QP2010 Plus equipped with a DB5-MS capillary column (Shimadzu, Nancy, France, with dimension: 30 m × 0.25 mm, film thickness 0.25 µm) was exploited. The injector and the detector were set at 250 °C. The oven temperature was programmed at 50 °C for 1 min, gradually increased to 250 °C at 5 °C/min. It was held for 10 min then increased later to 270 °C at 5 °C/min then held for 5 min. After that, it was increased to 280 °C at 5 °C/min and held for 10 more minutes. Although the split ratio was 10:1, the split flow was equal to 10 mL/min. In this process, helium was used as a carrier gas with a constant speed of 1.69 mL/min. The GC was also equipped with an Auto-Injector (Shimadzu, Nancy, France, AOC-5000) and the injected volume was equal to 1 µL. Mass units were monitored from 35 to 400 *m*/*z* at 70 eV. The mass spectra of the components were identified using data from the NIST Library (NIST08s).

### 2.4. Response Surface Methodology (RSM)

Response surface methodology was used to study the influence of supercritical operating extraction parameters such as CO_2_ pressure (*P*), CO_2_ flow rate ($Q_{{\mathrm{CO}}_{2}}$), and average particle size of the leaf (*d_P_*), on the extract oil yield. These three response variables were coded as *x*_1_, *x*_2_, and *x*_3_, respectively. The range and levels of independent factors were chosen based upon the results of preliminary tests and are gathered in Table 2. The individual and interactive effects of these parameters on the dependent variable were studied. Equation (1) represents the linear model with interactions for the three operating conditions,

(1) $$Y_{D}=a_{0}+a_{1}x_{1}+a_{2}x_{2}+a_{3}x_{3}+a_{12}x_{1}x_{2}+a_{13}x_{1}x_{3}+a_{23}x_{2}x_{3}$$

where Y_D_ represents a dependent variable (the yield of extract oil), *a*_0_ is a constant, *a*_1_, *a*_2_, and *a*_3_ are individual linear coefficient, *a*_12_, *a*_13_, and *a*_23_ are the interactive linear coefficient, and *x*_1_, *x*_2_, and *x*_3_ are the coded values of independent factors (pressure, CO_2_ flow rate, and average particle size respectively).

Nemrod-w software package was used for the regression analysis of the experimental data obtained [33]. Fit quality of the mathematical model equation was expressed by the determination coefficient R^2^, and its statistical significance was checked by an F-test. The significance of the regression coefficient was tested by a t-test. Significance level was given as *** *p* < 0.001, ** *p* < 0.01, * *p* < 0.05. Differences with *p*-value superior to 0.05 were not considered significant. For our experimental design validation, optimum conditions were fixed based on the data obtained from the experimental design.

### 2.5. Modeling of the Supercritical Extraction Process

Stastováet et al. [30] made several simplifications on Sovová’s model [29] by introducing two parameters: the grinding efficiency (G) and the dimensionless time (ψ),

(2) $$\psi =\frac{tQy_{s}}{Nx_{0}}$$

where *t* is the extraction time, *Q* represents the solvent mass flow rate, $y_{s}$ is the oil solubility in the solvent, *N* is the initial mass of the solid, and *x*_0_ is the initial oil concentration in the solid.

The mass of extracted oil (*E*) can be calculated by the following Equation (3)–(7):

(3) $$\frac{E}{N.x_{0}}=\{\begin{array}{c} \psi \left[1-\exp\left(-Z\right)\right]\mathrm{for}\;\psi <\frac{G}{Z}\\ \psi -\frac{G}{Z}\exp\left[Z\left(h_{k}-1\right)\right]\mathrm{for}\;\frac{G}{Z}\leq \;\psi <\psi_{k}\\ 1-\frac{1}{Y}\ln\left\{1+\left[\exp\left(Y\right)-1\right]\exp\left[Y\left(\frac{G}{Z}-\psi \right)\right]\left(1-G\right)\right\}\mathrm{for}\;\psi \geq \psi_{k} \end{array}$$

Inside the interval of dimensionless time, two regions exist between *G*/*Z* and ψ*_k_*, where

(4) $$\psi_{k}=\frac{G}{Z}+\frac{1}{Y}\ln\left\{1-G\left[1-\exp\left(Y\right)\right]\right\}$$

There is a region definition in dimensionless coordinate, namely *h_k_*.

(5) $$h_{k}=\frac{1}{Y}\ln\left[1+\frac{\left\{\exp\left[Y\left(\psi -\frac{G}{Z}\right)\right]-1\right\}}{G}\right]$$

The dimensionless quantities *Z* and *Y* are proportional to the mass transfer coefficients according to the first and second extraction period,

(6) $$Z=\frac{Nk_{f}a_{0}\rho_{f}}{Q\left(1-\varepsilon \right)\rho_{s}}$$

(7) $$Y=\frac{Nk_{s}a_{0}x_{0}}{Q\left(1-\varepsilon \right)y_{s}}$$

where $k_{f}$ and $k_{s}$ are the external and the internal mass transfer coefficients respectively, *a*_0_ is the particle specific interfacial area, $\rho_{f}$ stands for the solvent density, $\rho_{s}$ represents the solid density, and ε is the bed void volume.

The model was implemented in MATLAB^TM^. Three adjustable parameters were considered: the grinding efficiency (G), the internal mass transfer parameter ($k_{s}a_{0}$), and the external mass transfer parameter ($k_{f}a_{0}$). Equilibrium type “A” model was considered according to Sovová [28]. Since the external mass transfer parameter ($k_{f}a_{0}$) had no sensitivity [40,41], Fiori et al. [39] suggested an approach to determine this parameter referring to the literature correlations—Sherwood (Sh), Reynolds (Re) and Schmidt (Sc) numbers of experimental runs. CO_2_ physical properties were evaluated according to NIST database [42] and the oil extract properties were assumed to be related to the major compound found in GC-FID/MS analysis, α-pinene [43,44]. Binary diffusion coefficient (D_AB_) between the CO_2_ and the major compound was obtained by correlations. In the case of the supercritical fluid extraction, Sherwood number (Equation (8)) is a function of only Reynolds and Schmidt when natural convection is not significant [45,46,47],

(8) $$Sh=c_{0}Re^{c_{1}}Sc^{c_{2}}$$

where, $c_{0},\;c_{1}$ and $c_{2}$ are the adjustable parameters. According to the most-proposed correlations in the literature [45,46,47], $c_{0}$ should be higher than 1, $c_{1}$ is constrained between 0.5 and 0.8, and $c_{2}=1/3$.

Catchpole et al. [48] and Lito et al. [49] used the approach to estimate only two adjustable parameters (G, and $k_{s}a_{0}$) directly using the experimental kinetic curve, and they calculated the other parameter ($k_{f}a_{0}$) using the Sherwood correlation because they considered that it was not significant. However, in our approach, we estimated not only these two parameters (G and $k_{s}a_{0}$), but also the external mass transfer parameter ($k_{f}a_{0}$) using the experimental curve for more accuracy.

The relevance of the model fitting to the experimental data was assessed considering two statistical criteria, namely the coefficient of determination R^2^ detrmined using Equation (9) and the root means square error (RMSE) given by Equation (10),

(9) $$R^{2}=\;1-\frac{\sum_{i}^{n}{\left(y_{\left(i\right)exp}-y_{\left(i\right)model}\right)}^{2}}{\sum_{i}^{n}{\left(y_{\left(i\right)exp}-\bar{y_{exp}}\right)}^{2}}$$

(10) $$RMSE=\;\sqrt{\overset{n}{\underset{i}{\sum }}\frac{{\left(y_{\left(i\right)exp}-y_{\left(i\right)model}\right)}^{2}}{n}}$$

where *n* represents the number of available experimental data, and $y_{exp}$ and $y_{model}$ are the experimental extraction yield and the extraction yield predicted by the model, respectively.

### 2.6. Cost Estimation of Processes and Scale-Up

The manufacturing cost of the supercritical extract was estimated through methodology proposed elsewhere [50,51]. Concerning material cost, electricity and labor, they were collected from regional information in Tunis (Tunisia 2016). The fixed investment cost was obtained from the literature proposed by Turton et al. [52] to evaluate the cost of manufacturing according to Equation (11) including depreciation (10% of FCI).

(11) $$COM=0.340FCI+2.73C_{OL}+1.23\left(C_{UT}+C_{WT}+C_{RM}\right)$$

where *COM* is the cost of manufacturing of supercritical extract of *Pistacia*, FCI corresponds to the fixed cost of investment, *C_OL_* represents the cost of operational labor, *C_UT_* is the cost of utilities, *C_WT_* the cost of waste treatment, and *C_RM_* is the cost of raw material.

According to Carvalho [53], Equation (12) can be used in order to determine the solvent flow rate required to maintain the same kinetic behavior in different SFE units (scale-up) for a given feed mass and bad geometry. Researchers [54,55,56,57] declared that the extraction time has an influence on an extraction’s *COM* and the extraction rate increases by increasing the solvent flow rate. They also reported that oil yield in the extraction can be positively influenced by the solvent flow rate increases as the following equation:

(12) $$\frac{Q_{{\mathrm{CO}}_{2}}^{2}}{Q_{{\mathrm{CO}}_{2}}^{1}}={\left(\frac{F_{2}}{F_{1}}\right)}^{2}\left(\frac{H_{B1}}{H_{B2}}\right)\left(\frac{d_{B1}}{d_{B2}}\right)$$

The extractor geometry data and the installed supercritical extraction cost were obtained from Núñez and del Valle [58,59,60]. In fact, a plant with two extraction vessels, each one having internal volume varying between 0.2, 0.4, 0.6 and 1.0 m^3^, was evaluated. For instance, for a plant with vessels of 1.0 m^3^, the aspect ratio was H/d = 8 (with 0.542 m × 4.334 m of inner diameter and height respectively). The wall thickness withstands 390 bar. The fixed cost of investment (FCI) of each SFE unit was determined in USD based on the values of Rocha-Uribe et al. [59]. All the values are reported in Table 3 and are calculated using the Chemical Engineering Plant Cost Index (CPECI) value for 2014 (CPECI 2014 = 580) [61].

According to Experiment (2), the relations of laboratory-scale H/d is 3.6, with the flow rate from 3.36 × 10^−5^ kg/s, the bed apparent density ρ = 296 kg/m^3^, with the operational conditions of 220 bar and 40 °C for the extraction process were taken into consideration.

**Figure 2:**
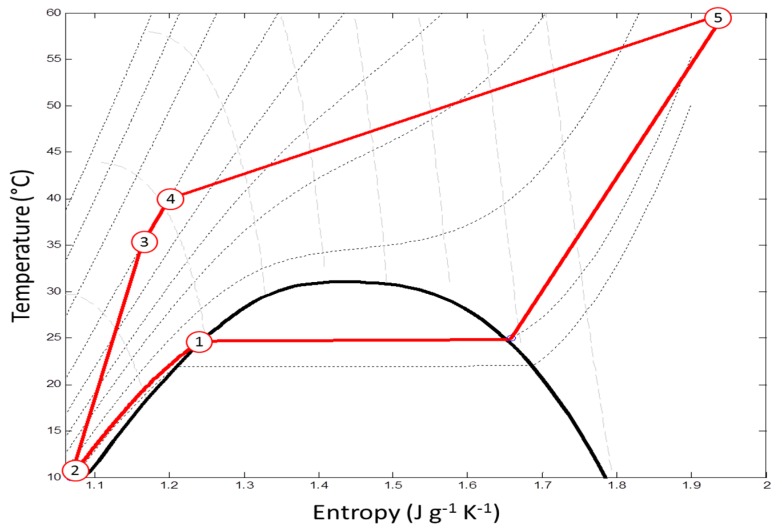
Entropy (s) diagram of CO_2_ cycle during supercritical fluid extraction. Circled number 1 represents saturated liquid at 25 °C and 64 bar; 2 is pump inlet (10 °C and 64 bar); 3 is pump exit (35 °C and 220 bar); 4 is extraction vessels (40 °C and 220 bar); 5 is separation vessel (60 °C and 60 bar).

Figure 2 shows the cycle of solvent (pure CO_2_) during the supercritical extraction process in an operating unit. The steps are considered as being primarily solvent collection in reservoir (64 bar and 25 °C), followed by a cooling process (10 °C), pumping and pressurization of the extraction vessel (220 bar and 35 °C), followed by temperature increase (40 °C) until obtaining the desired extraction condition and finally, after this process, reducing the pressure (60 bar and 60 °C) for solute precipitation for reuse.

In this case, it is assumed that during the process of decompression, the solute is separated, and the pure solvent is returned to the system. Based on the findings of this and the mutual values found in other research papers, it is identified that extraction yields (mass extract/*Pistacia* load) were estimated to reach 0.3%, 0.5%, 0.7%, 1.0% and 1.5%.

The cost of 8000 h per year operational work, with continuous 24 h per day, 8 h daily shifts (2 workers/shift) was considered. The cost of labor was operationally considered to be 6.60 USD/h (1475.60 USD/month tax included). The utility cost was estimated relying on energy consumption involved in the solvent cycle CO_2_, cold water, and electricity [51,58]. The specific energies of CO_2_ for cooling, heating, and pumping in the solvent cycle were equal to −261.29 kJ/kg, 219.2 kJ/kg and 55.0196 kJ/kg, respectively. These calculations were based upon the work of Rock-Uribe et al. [59].

The electricity cost was equal to 217.10 USD/MWh (price charged in Tunis, Tunisia, tax included). Concerning raw material costs, the considered values were: 1.35 USD/kg of dried and milled *Pistacia* leaves, 0.15 USD/kg of CO_2_ and 0.97 USD/kg of ethanol for cleaning purposes [60]. It was considered that 2% of CO_2_ mass was lost during the extraction cycle for all the SFE process scale evaluated in this work. The cost of waste treatment was not considered because CO_2_ was fully recycled and *Pistacia* leaf can be used in soil enrichment or energy generation.

## 3. Results and Discussion

Table 4 shows the experimental yield results and operating conditions of supercritical extraction for the ten experiments. The experimental oil extract yield was calculated using the following Equation (13):

(13) $$Yield\;\left(\mathrm{wt}\%\right)=\frac{Mass\;of\;leaf\;extract}{Mass\;of\;raw\;material}\times 100$$

The yield observed for the tested conditions varied between 0.093% and 0.285%. We found that Experiment (2) with the highest pressure, lowest flow rate, and the lowest average particle diameter gave the greatest tested income extraction conditions. The replications performed at 80 bars (0.10% ± 0.0184%), which were Experiment (3) and (4), demonstrated higher variability than those performed at 180 bar (0.022% ± 0.0057%) (Experiments (8) and (9)); therefore, they have coefficients of variation equal to 17.3% and 2.5% respectively.

By comparing all results, we observe that the present work provided different yields that were in some cases inferior to those reported by other authors [31,60]. In fact, Bampouli et al. [62] obtained an outcome from the leaves of *Pistacia lentiscus* (PL) var. chia (from Chios, Greece) varying between 1.6% and 5% *w*/*w*. The conditions were ranging from 100 to 250 bar and 45 °C with a flow rate of 1.5 to 3.0 kg CO_2_/h. Also, Congiu et al. [31] acquired yields between 0.25% and 0.45% for the leaves of *Pistacia lentiscus* (PL) coming from the regions of Costa Rey and Capoterra (Sardinia, Italy) providing 90 bar and 50 °C with a flow rate of 0.9 kg CO_2_/h.

The variation of the obtained yields must be due to the areas of cultivation processing, treatment of raw materials and experimental conditions in the extraction process. Appendix A shows the characterizations of the essential oil obtained from the leaves and carried out using GC-MS-FID. We observed that the major compounds for the extraction of *Pistacia lentiscus* in the Tunis region are α-pinene (32%), followed by terpinene-4-ol (13%), 1-8-cineole (6%), α-terpineol (4%), β-caryophyllene (4%) and borneol (4%), as summarized in Table 5. Furthermore, as expected, these compositions are not significantly influenced by changing the operating conditions due to the constant extraction temperature.

### 3.1. Study the Effect of Operating Conditions on the Yield Using RSM

Response surface methodology (RSM) was used to study the individual and the interactive influence of operating extraction parameters on the extract yield to find the optimal operating conditions. Table 6 shows the experimental design yield for the ten experiments.

**Figure 3:**
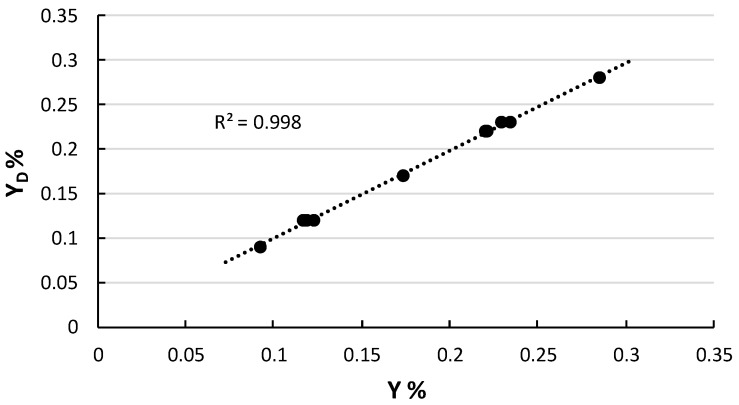
Experimental design yield (Y_D_) versus experimental yield (Y).

We used the analysis of variance (ANOVA) to evaluate the statistical significance of the linear model represented in Equation (1). The model can describe the variation of the results because it is significant at <5%. We, also, verified the model efficiency and the adaptability to the experimental data by estimating the coefficient of actual and predicted determination (R^2^ and predicted R^2^ respectively) calculated by the analysis of variance. We found out that the actual determination coefficient indicates that the fitted model explains 91.2% of the variability in the extraction yield. The predicted R^2^ was 0.998 (a good agreement) indicating that our experimental design can be used for modeling the response variables employed, as shown in Figure 3.

Table 7 gathers the statistical results of the constant parameters in Equation (1): the linear intercept constant (*a*_0_), the individual linear effects of the three independent variables (*a*_1_, *a*_2_, and *a*_3_), and their interactive linear effects (*a*_12_, *a*_13_, and *a*_23_). Therefore, the linear regression equation used to evaluate the experimental yield becomes (Y_D_),

(14) $$Y_{D}=0.183+0.084x_{1}$$

This equation indicates that the main factor that significantly influences the yield was the CO_2_ pressure when the confidence of 5% was considered. The equation identifies the best conditions through variation of chosen parameters to maximize the extraction efficiency, which is presented in Experiment (2).

**Figure 4:**
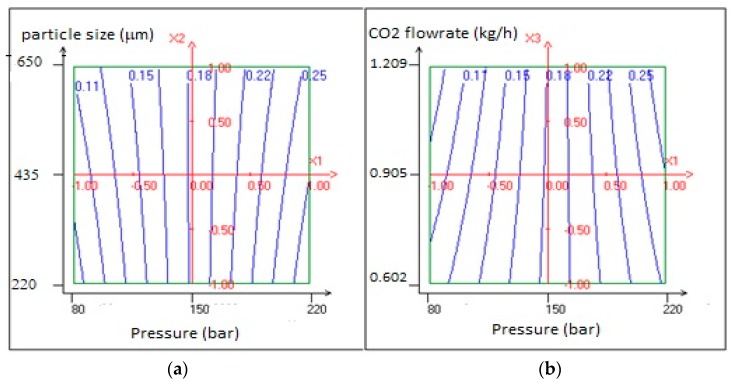
Surface plots of the experimental design yield as a function of (**a**) CO_2_ pressure and average particle size (**b**) CO_2_ pressure and CO_2_ flowrate.

For a better understanding of the statistical results, Figure 4 represents the 2D response surface of the experimental yields by the function of pressure, average particle size and the flow rate of CO_2_. As can be observed in Figure 4, the highest yield was obtained around the maximum point of pressure (*P* = 220 bar) when the flow rate of CO_2_ and average particle size were around minimum points.

### 3.2. Analysis and Validation of Experimental Design

The statistical analysis for the final selected model shows that the effect of the CO_2_ pressure is the only variable that has a significant effect on the yield, compared to the other variables that have no effects. For this reason, we analyzed the selection effect of the identification and validation points used for our experimental design. The existence of a correlation between the parameters increases the size of the confidence intervals [63], therefore we need to control the value of the correlation coefficients 2 to 2.

In our investigation, we have used the D-optimality criterion [64] to separate the experiments used for both parametric identification and validation model. This method consists of choosing a set of parametric identification points to obtain the highest determinant of the Fischer matrix information [65]. To investigate the influence of selection on the confidence intervals of each parameter, we studied the correlations between the parameters using the following matrix correlation coefficients summarized in Table 8.

**Figure 5:**
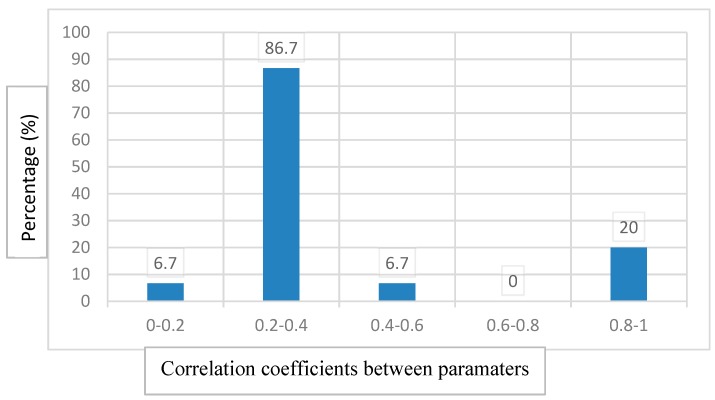
Percentage of correlation coefficients between paramaters.

Figure 5, which represents the frequency of the correlation coefficient, mimics 87% of the correlation between the parameters pairs are in the range of 0.2 to 0.4. This indicates the use of the Detmax Fedrov algorithm [66] that has no effect on the correlation between the parameters. Therefore, the used supports of a linear model with interaction, and subsequently the experimental design, are applicable at least in this study.

### 3.3. Effect of Operating Conditions on the Mass Transfer

All experimental data were used to determine the model parameters (G, and $k_{s}a_{0}$, and $k_{f}a_{0}$) (see Appendix B). Table 9 shows that the two adjusted parameters (grinding efficiency (G) and internal mass transfer parameter ($k_{s}a_{0}$), which is estimated by the experimental kinetic curves, have no significant change between the two approaches. As expected [3,13,31], the value of grinding efficiency (G) increases by decreasing the average particle size. This parameter is not only related to the particle size, but also to the shape of its distribution (normal, bimodal). As a result, it influences the curve shape due to the solvent flow asymmetry effect.

In the first approach, we applied the correlations proposed by Lito, Catchpole, and King using only the binary diffusion coefficient (CO_2_-α-pinene) to estimate the external mass transfer parameter ($k_{f}a_{0}$). On the other hand, the adjusted parameters were not only grinding efficiency (G) and the internal mass transfer parameter ($k_{s}a_{0}$) but also the external mass transfer parameter, which was estimated by experimental extraction curves in the second approach.

Table 10 shows the parameters ($k_{f}a_{0}$, and D_AB_) for both approaches. The adjusted parameter ($k_{f}a_{0}$) estimated by the experimental kinetic curves in the second approach is different than the correlated parameter proposed by Lito and Catchpole in the first approach. This parameter affects the values of the diffusion coefficient as shown in Table 10.

In the second approach, the parameter values of the Sherwood number were experimentally obtained from the extraction curves and *k_f_* values. The parameter correlation suggested in the second approach was determined from *c*_0_ and *c*_1_ settings, resulting in the equation as shown in Table 11.

(15) $$Sh=0.0349Re^{0.58}Sc^{1/3}$$

The adjusted parameters are within the range reported by [19] and their applicability for Reynolds and Schmidt values are in the ranges 2 ≤ Re ≤ 60 and 2 ≤ Sc ≤ 12.

The values of the mass transfer coefficients ($k_{f}a_{0}$) ranged from 1.4 × 10^−2^ to 3.7 × 10^−1^ for the estimation performed with the correlations of Lito and Catchpole.

Concerning the adjustment made with the three parameters G, $k_{f}a_{0}$ and $\;k_{s}a_{0}$ (the value of $k_{f}a_{0}$ is 0.020), it ranged from 7.3 × 10^−3^ s^−1^ to 8.9 × 10^−2^ s^−1^. These values are lower than those obtained by adjustment with the correlations. The parameters $k_{s}a_{0}$ were in all cases between 1.9 × 10^−5^ s^−1^ and 1.8 × 10^−4^ s^−1^.

### 3.4. Cost Estimation of Processes and Scale-Up

**Figure 6:**
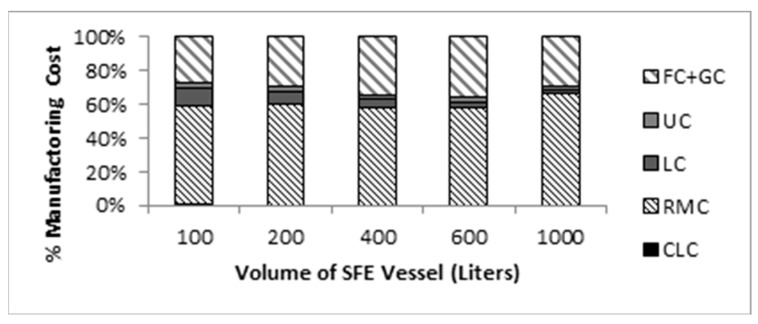
Each cost category in the manufacturing cost of *Pistacia lentiscus* supercritical extract.

The greatest impact on the cost of manufacturing extracted oil production of *P. lentiscus* in Tunisia is represented by the raw material cost (RMC), followed by the fixed cost of investment and the utility cost, as indicated in Figure 6.

Table 12 shows the manufacturing costs of the supercritical extract of *Pistacia lentiscus* in the US. We note that for the same yield, the lowest costs were those obtained at the highest production volume, as was expected.

Concerning yield values obtained on a pilot scale (0.30%), the cost of the manufacturing process of the supercritical extract weas between 999.63 USD/kg and 813.95 USD/kg. These values are considered very high when compared to the costs of other vegetable raw materials such as rosemary extract which is worth 49.71 USD/kg [21], ginger oleoresin which costs 99.80 USD/kg [20], *Curcuma longa* L. extract which is worth 164.4 USD/kg [40] and habanero pepper extract with a cost of 540.19 USD/kg [33].

A yield increase in the extraction process reduces the cost of manufacturing. Thus, the high cost of production is due to the low yields gained from this kind of plant. Prices of traded extracted oils of *Pistacia lentiscus* sold in 5 mL, 10 mL or 30 mL vials are about 5.83 USD/g (5830.00 USD/kg). These values are obtained from the local market in Tunis, Tunisia.

## 4. Conclusions

*Pistacia lentiscus* L. plant from the Tunisian region is appeased of medicinal properties in its extract oil that can be produced using supercritical carbon dioxide (SC-CO_2_) extraction. In this study, we observed that the α-pinene (32%) was the major compound of the extracted oil of *Pistacia lentiscus* in the Tunis region. The experiment (2) having the highest pressure, lowest flow rate, and the lowest average particle diameter gave the greatest tested income extraction conditions.

We investigated the influence of CO_2_ pressure, average particle size, and CO_2_ flow rate and their interaction on the extract yield using the response surface methodology (RSM). It was observed that the main factor that significantly influences the yield was the CO_2_ pressure and, therefore, the best conditions through variation of chosen parameters to maximize the extraction efficiency were presented in Experiment (2).

We studied the influence of operating parameters on mass transfer by evaluating a process applying broken and intact cell (BIC) on the essential oil extraction curves that are acquired from the leaves of *Pistacia lentiscus* L. The two adjusted parameters (grinding efficiency (G) and internal mass transfer parameter ($k_{s}a_{0}$)), which were estimated by the experimental kinetic curves, have no significant change between the two approaches (Lito and Catchpole approach, and AYDI A approach). However, the external mass transfer parameter ($k_{f}a_{0}$) proposed by AYDI A was different from the correlated parameter in the first approach that significantly influences the values of the diffusion coefficient.

The economic evaluation in the scale-up process was obtained for the SC-CO_2_ extraction of these plant leaves. We indicated that the lowest costs were obtained at the highest production volume for the same yield. The manufacturing cost of oil production is reduced by a yield increase in the extraction process because of the low yields obtained from this type of plant.

## Figures and Tables

**Table 1:** Conditions of supercritical fluid extraction.

Experiments	*P* [bar]	*d_P_* [µm]	$Q_{CO_{2}}$ [kg h^−1^] *	$\rho_{CO_{2}}$ [kg m^−3^]	$\mu_{CO_{2}}\cdot 10^{5}$ [kg m s^−1^]
1	220	650	0.604	857.20	8.18
2	220	220	0.602	857.20	8.18
3	80	650	1.202	277.90	2.23
4	80	650	1.209	277.90	2.23
5	80	220	0.602	277.90	2.23
6	80	650	0.603	277.90	2.23
7	140	650	1.204	763.27	6.51
8	180	220	0.913	819.51	7.45
9	180	220	0.904	819.51	7.45
10	180	650	0.908	819.51	7.45

* Deviation ± 0.03 kg h^−1^ of CO_2_.

**Table 2:** Conditions of supercritical fluid extraction.

Variable	Symbol	Factor Level	
		−1	1
Pressure (bar)	*x*_1_	80	220
CO_2_ flowrate (kg/h)	*x*_2_	0.6	1.2
Average particle size (µm)	*x*_3_	220	650

**Table 3:** Estimated cost of each supercritical fluid extraction (SFE) unit, including all equipment (Chemical Engineering Plant Cost Index, CPECI, 2014 = 580).

Extractor Vessel (liters)	H (m)	d (m)	H/d	Fixed Cost (FCI) (US $)
**100**	2.01	0.252	8.0	$853,975
**200**	2.54	0.317	8.0	$1,378,550
**400**	3.19	0.399	8.0	$2,225,400
**600**	3.66	0.457	8.0	$2,944,900
**1000**	4.34	0.542	8.0	$4,191,250

**Table 4:** The yield of supercritical extraction, with three different variables (*P*, *dp* and $Q_{{\mathrm{CO}}_{2}}$ ).

Experiment	*x*_1_ *P* [bar]	*x*_2_ *d_P_.* [µm]	*x*_3_ $Q_{CO_{2}}\;[\mathrm{kg}\;h{}^{-1}]$	Yield [%]
1	220	650	0.604	0.234
2	220	220	0.602	0.285
3	80	650	1.202	0.093
4	80	650	1.209	0.119
5	80	220	0.602	0.123
6	80	650	0.603	0.117
7	140	650	1.204	0.221
8	180	220	0.913	0.220
9	180	220	0.904	0.229
10	180	650	0.908	0.174

**Table 5:** Areas of compounds found in the oils obtained by SFE from leaves.

Compounds	RI	Exp.1	Exp.2	Exp.3	Exp.4	Exp.5	Exp.6	Exp.7	Exp.8	Exp.9	Exp.10
α-pinene	**939**	33.30	30.00	34.21	30.10	33.21	32.10	34.31	31.10	32.41	32.1
Terpinene-4-ol	**1178**	13.04	13.24	13.08	13.68	13.02	12.01	12.77	12.06	13.12	12.16
1-8-cineole	**1033**	5.10	6.10	6.02	6.62	5.85	6.42	5.66	6.11	5.06	7.11
α-terpineol	**1189**	4.61	4.01	4.58	4.88	4.12	4.21	4.06	4.67	4.68	4.55
β-caryophyllene	**1434**	4.02	4.92	4.22	4.82	4.88	4.03	4.12	4.43	4.01	4.93
Borneol	**1165**	3.92	3.12	4.62	4.02	4.22	4.12	4.45	4.16	4.85	4.66
Others		36.01	38.61	31.27	35.88	34.70	37.11	34.63	37.47	35.87	34.49

**Table 6:** Design yield (Y_D_) for supercritical extraction of *Pistacia lentiscus*.

Experiment	*x*_1_	*x*_2_	*x*_3_	Y_D_ (%)
**1**	1.0000	1.0000	−0.9934	0.23
**2**	1.0000	−1.0000	−1.0000	0.28
**3**	−1.0000	1.0000	0.9769	0.09
**4**	−1.0000	1.0000	1.0000	0.12
**5**	−1.0000	−1.0000	−1.0000	0.12
**6**	−1.0000	1.0000	−0.9967	0.12
**7**	−0.1429	1.0000	0.9835	0.22
**8**	0.4286	−1.0000	0.0247	0.22
**9**	0.4286	−1.0000	−0.0049	0.23
**10**	0.4286	1.0000	0.0082	0.17

**Table 7:** Coefficient of a linear regression equation (Equation (1)).

Coefficient	Coefficient Value	Test Experiment	*p*-Value along with Confidence %
***a*_0_**	0.183	12.43	***
***a*_1_**	0.084	3.71	*
***a*_2_**	0.000	0.01	99.1%
***a*_3_**	−0.002	−0.10	92.2%
***a*_12_**	−0.013	−0.80	48.5%
***a*_13_**	0.017	0.71	53.3%
***a*_23_**	0.019	0.86	45.3%

* *p* < 0.05; *** *p* < 0.001.

**Table 8:** Matrix correlation coefficients.

Parameters	*a*_1_	*a*_2_	*a*_3_	*a*_12_	*a*_13_	*a*_23_
*a*_1_	1.0000	−0.3039	−0.3291	0.2608	−0.3623	−0.2981
*a*_2_	−0.3039	1.0000	0.3909	−0.0455	−0.2335	−0.2095
*a*_3_	−0.3291	0.3909	1.0000	−0.3748	−0.3771	0.4994
*a*_12_	0.2608	−0.0455	−0.3748	1.0000	0.2565	−0.2247
*a*_13_	−0.3623	−0.2335	−0.3771	0.2565	1.0000	−0.2563
*a*_23_	−0.2981	−0.2095	0.4994	−0.2247	−0.2563	1.0000

**Table 9:** The adjusted parameters (G and *k_s_a*_0_) between two approaches.

Exp.	*P* [bar]	*d_P_* [µm]	$Q_{CO_{2}}$ [kg.h^−1^]	G			*k_s_a*_0_.10^5^ (s^−1^)		
				1st App.		2nd App.	1st App.		2nd App.
				Lito	Catchpole	AYDI A.	Lito	Catchpole	AYDI A.
**1**	220	650	0.604	0.36	0.36	0.37	7.48	7.48	7.35
**2**	220	220	0.602	0.61	0.61	0.61	7.01	7.01	7.01
**3**	80	650	1.202	0.39	0.39	0.40	0.182	0.182	0.182
**4**	80	650	1.209	0.36	0.36	0.37	0.107	0.107	0.107
**5**	80	220	0.602	0.52	0.52	0.52	9.28	9.28	9.30
**6**	80	650	0.603	0.44	0.44	0.45	7.58	7.58	7.58
**7**	140	650	1.204	0.35	0.35	0.37	7.83	7.84	7.71
**8**	180	220	0.913	0.62	0.62	0.66	2.75	2.75	2.31
**9**	180	220	0.904	0.54	0.54	0.59	2.32	2.32	1.91
**10**	180	650	0.908	0.23	0.23	0.23	0.108	0.108	0.108

**Table 10:** Parameters (*k_f_a_0_*, and D_AB_) between two approaches.

Exp.	*P* [bar]	*d_P_* [µm]	$Q_{CO_{2}}$ [kg.h^−1^]	*k_f_a*_0_.10^3^ (s^−1^)			*D_AB_*. 10^9^(m^2^ s^−1^)		
				1st App.		2nd App.	1st App.		2nd App.
				Lito	Catchpole	AYDI A.	Lito	Catchpole	AYDI A.
**1**	220	650	0.604	1.41	1.46	0.736	8.63	9.06	2.97
**2**	220	220	0.602	6.56	6.77	1.72	8.63	9.06	1.06
**3**	80	650	1.202	0.114	0.118	8.70	38.0	40.2	0.73
**4**	80	650	1.209	0.114	0.119	8.95	38.0	40.2	0.76
**5**	80	220	0.602	0.355	0.369	3.42	38.0	40.2	1.04
**6**	80	650	0.603	7.63	7.93	4.03	38.0	40.2	13.3
**7**	140	650	1.204	2.67	2.77	1.26	10.7	11.3	3.15
**8**	180	220	0.913	9.19	9.51	0.773	9.4	9.9	6.61
**9**	180	220	0.904	9.14	9.46	0.929	9.4	9.9	8.79
**10**	180	650	0.908	1.97	2.04	2.64	9.4	9.9	13.4

**Table 11:** Number and the coefficient of determination r^2^ between two approaches.

Exp.	*P* [bar]	*d_P_* [µm]	$Q_{CO_{2}}$ [kg.h^−1^]	Sh			r^2^		
				1st App.		2nd App.	1st App.		2nd App.
				Lito	Catchpole	AYDI A.	Lito	Catchpole	AYDI A.
**1**	220	650	0.604	0.24	0.24	0.96	98.478	98.477	98.472
**2**	220	220	0.602	0.13	0.13	4.16	99.352	99.352	99.355
**3**	80	650	1.202	0.45	0.44	0.06	99.716	99.716	99.718
**4**	80	650	1.209	0.45	0.44	0.05	99.632	99.632	99.626
**5**	80	220	0.602	0.16	0.16	1.24	99.297	99.297	99.357
**6**	80	650	0.603	0.30	0.30	0.13	98.302	98.301	98.322
**7**	140	650	1.204	0.37	0.37	0.40	98.548	98.541	98.663
**8**	180	220	0.913	0.17	0.16	5.84	98.268	98.268	99.137
**9**	180	220	0.904	0.17	0.16	5.11	98.421	98.421	99.019
**10**	180	650	0.908	0.31	0.31	0.29	99.381	99.381	99.381

**Table 12:** Manufacturing cost of a supercritical extract of *Pistacia lentiscus* leaves, in a batch of 60 min (220 bar and 40 °C).

Volume (m^3^)	*COM* (US$/kg)				
	Expected Oil Yield (%)				
	0.3	0.5	0.7	1.0	1.5
**0.1**	999.63	599.78	428.41	299.89	199.93
**0.2**	942.63	565.58	403.99	282.79	188.53
**0.4**	952.04	571.22	408.02	285.61	190.41
**0.6**	945.12	567.07	405.05	283.53	189.02
**1.0**	813.95	488.37	348.83	244.18	162.79

## Appendix A

**Table A1:** Percentages areas of compounds found in the oils obtained by SFE from leaves in each experiment.

Compounds	RI	Exp.1	Exp.2	Exp.3	Exp.4	Exp.5	Exp.6	Exp.7	Exp.8	Exp.9	Exp.10
Tricyclene	**924**	1.05	0.9	0.48	0.58	0.48	1.2	0.75	0.64	0.46	0.14
**α-pinene**	**939**	33.3	30.0	34.21	30.1	33.21	32.1	34.31	31.1	32.41	32.1
Z-3-hexenol	**855**	0.12	0.15	0.32	0.22	0.23	0.11	0.86b	0.82	0.64	0.32
E-2-hexenol	**856**	0.09	0.01	0	0	0	0	0.033	0.08	0.03	0.01
Hexanol	**865**	0.25	0.36	0.31	0.51	0.41	0.45	0.63	0.84	0.65	0.8
E-2-hexenal	**850**	0.19	0.12	0.28	0.48	0.58	0.58	0.65	0.67	0.91	0.56
α-thujene	**928**	0.2	0.21	0.41	0.21	0.31	0.24	0.56	0.58	0.56	0.88
Camphor	**954**	1.6	1.8	1.45	1.05	1.65	1.15	1.66	1.53	2.06	1.43
xxCamphene	**954**	0.89	*	1.2	1.02	1.42	1.12	1.22	1.08	1.02	0.66
Sabinene	**975**	0.19	0.22	0.22	0.32	0.42	0.22	0.56	0.13	0.64	0.23
β-pinene	**980**	2.04	2.54	2.17	2.07	2.37	2.17	2.81	2.83	2.25	2.12
Myrcene	**991**	0.24	0.26	0.17	0.27	0.37	0.17	0.4	0.24	0.3	0.14
α-phellandrène	**1006**	0.24	0.25	0.3	0.22	0.32	0.28	0.24	0.14	0.18	0.24
∆-3-carene	**1011**	0.22	0.12	0	0	0	0	0	0	0	0
**p-cymene**	**1026**	1.7	2.7	3.04	3.54	2.95	3.84	2.6	3.01	2.05	3.11
Limonene	**1030**	1.19	1.89	2.01	2.41	2.21	2.51	2.02	1.55	2.12	1.95
1-8-cineole	**1033**	5.1	6.1	6.02	6.62	5.85	6.42	5.66	6.11	5.06	7.11
(*E*)-β-Ocimene	**1050**	0.52	0.82	0.82	0.62	0.92	0.72	0.78	0.62	0.44	0.44
γ-terpinene	**1053**	0.5	0.66	0.56	0.36	0.33	0.56	0.12	0.12	0.18	0.44
Oxyde de Cis-linalool	**1074**	0.94	1.4	1.79	1.49	1.09	1.44	1.72	1.68	1.02	1.38
Oxyde de Trans linalool	**1088**	0.6	0.8	0.98	0.58	0.88	0.58	0.85	0.66	0.75	0.76
Terpinolene	**1092**	0.06	0.16	0.48	0.68	0.58	0.74	0.64	0.92	0.2	0.82
Linalool	**1098**	2.59	2.89	2.89	2.29	2.69	2.19	2.94	2.06	2.86	2.26
Borneol	**1165**	3.92	3.12	4.62	4.02	4.22	4.12	4.45	4.16	4.85	4.66
**Terpinene-4-ol**	**1178**	13.04	13.24	13.08	13.68	13.02	12.01	12.77	12.06	13.12	12.16
**α-terpineol**	**1189**	4.61	4.01	4.58	4.88	4.12	4.21	4.06	4.67	4.68	4.55
Geraniol	**1255**	0.69	0.89	0.79	0.99	0.59	0.59	0.69	0.4	0.55	0.65
Acetate de bornyle	**1295**	2.82	2.12	2.02	2.12	2.01	2.19	2.78	2.85	2.08	2.15
Tridecane	**1300**	0.08	0.08	0.03	0.05	0.05	0.06	0.04	0.06	0.03	0.05
Linalyl de proprionate	**1325**	1.55	1.55	1.95	1.85	1.55	2.01	1.84	1.27	1.12	1.2
Acetate d’α terpenyle	**1344**	0.73	0.73	0.63	0.69	0.77	0.74	0.88	0.98	0.18	0.48
α-cubebene	**1351**	0.04	0.04	0.26	0.33	0.46	0.53	0.77	0.67	0.47	0.7
Copaene	**1372**	0.77	0.96	0.86	0.66	0.85	0.67	0.66	0.61	0.44	0.81
Β-elemene	**1391**	0.83	0.78	0.93	0.9	1.2	1.1	1.43	1.31	1.13	1.41
**β-caryophyllene**	**1434**	4.02	4.92	4.22	4.82	4.88	4.03	4.12	4.43	4.01	4.93
α-humulene	**1454**	0.64	0.64	0.29	0.31	0.25	0.42	0.43	0.71	0.12	0.91
Allo-aromandrene	**1474**	0.51	0.11	0.21	0.31	0.21	0.61	0.24	0.51	0.41	0.15
Delta muurolene	**476**	0.11	0.41	0.66	0.65	0.86	0.78	0.94	0.31	0.44	0.39
GermacreneD	**1480**	0.12	0.31	0.72	0.77	0.62	0.84	0.91	0.55	0.77	0.65
Nonadecanone	**1900**	0.04	0.08	0	0	0	0	0.02	0.04	0	0.05

* Not identified compound.

## Appendix B

**Table A2:** Operating conditions estimated TWO parameters from best fitting and modeling errors used by Lito.

Experiment	*P* (bar)	$Q_{{\mathrm{CO}}_{2}}\;(\mathrm{kg}\;h^{-1})$	*d_P_*.10^4^ (m)	G (dimensionless)	*k_f_a*_0_ 10^2^ (s^−1^)	*k_s_a*_0_ 10^5^ (s^−1^)	*y_s_* 10^4^ (kg kg^−1^)	*k_f_* 10^6^ (m s^−1^)	*k_s_* 10^9^ (m s^−1^)	*D_AB_* 10^9^ (m^2^ s^−1^)	Sh	Re	Sc	r^2^	RMSE 10^2^
**1**	220	0.604	6.5	0.36	1.41	7.48	4.1	3.25	0.172	8.63	0.24	7.33	11.06	98.478	3.73
**2**	220	0.602	2.2	0.61	6.56	7.01	5.2	5.12	5.47	8.63	0.13	2.47	11.06	99.352	2.41
**3**	80	1.202	6.5	0.39	0.114	0.182	0.9	0.263	0.420	0.38	0.45	53.53	2.11	99.716	1.73
**4**	80	1.209	6.5	0.36	0.114	0.107	0.9	0.263	0.246	0.38	0.45	53.83	2.11	99.632	1.96
**5**	80	0.602	2.2	0.52	0.355	9.28	2.2	0.277	7.24	0.38	0.16	9.08	2.11	99.297	2.62
**6**	80	0.603	6.5	0.44	7.63	7.58	2.6	0.176	0.175	0.38	0.30	26.84	2.11	98.302	3.77
**7**	140	1.204	6.5	0.35	2.67	7.83	1.5	6.16	0.180	0.107	0.37	18.37	7.98	98.548	3.85
**8**	180	0.913	2.2	0.62	9.19	2.75	2.4	7.17	2.15	9.40	0.17	4.12	9.67	98.268	3.37
**9**	180	0.904	2.2	0.54	9.14	2.32	2.9	7.13	1.81	9.40	0.17	4.08	9.67	98.421	3.00
**10**	180	0.908	6.5	0.23	1.97	0.108	1.8	4.53	2.49	9.40	0.31	12.10	9.67	99.381	2.73

**Table A3:** Operating conditions estimated TWO parameters from best fitting and modeling errors used by Catchpole.

Experiment	*P* (bar)	$Q_{{\mathrm{CO}}_{2}}\;(\mathrm{kg}\;h^{-1})$	*d_P_*.10^4^ (m)	G (dimensionless)	*k_f_a*_0_ 10^2^ (s^−1^)	*k_s_a*_0_ 10^5^ (s^−1^)	*y_s_* 10^4^ (kg kg^−1^)	*k_f_* 10^6^ (m s^−1^)	*k_s_* 10^9^ (m s^−1^)	*D_AB_* 10^9^ (m^2^ s^−1^)	Sh	Re	Sc	r^2^	RMSE 10^2^
**1**	220	0.604	6.5	0.36	1.46	7.48	4.1	3.36	0.172	9.06	0.24	7.33	10.54	98.477	3.73
**2**	220	0.602	2.2	0.61	6.77	7.01	5.2	5.29	5.47	9.06	0.13	2.47	10.54	99.352	2.41
**3**	80	1.202	6.5	0.39	0.118	0.182	0.9	0.273	0.420	0.402	0.44	53.53	2.00	99.716	1.73
**4**	80	1.209	6.5	0.36	0.119	0.107	0.9	0.274	0.247	0.402	0.44	53.83	2.00	99.632	1.96
**5**	80	0.602	2.2	0.52	0.369	9.28	2.2	0.288	7.24	0.402	0.16	9.08	2.00	99.297	2.62
**6**	80	0.603	6.5	0.44	7.93	7.58	2.6	0.183	0.175	0.402	0.30	26.84	2.00	98.301	3.77
**7**	140	1.204	6.5	0.35	2.77	7.84	1.5	6.39	0.180	0.113	0.37	18.37	7.55	98.541	3.85
**8**	180	0.913	2.2	0.62	9.51	2.75	2.4	7.42	2.15	9.90	0.16	4.12	9.18	98.268	3.37
**9**	180	0.904	2.2	0.54	9.46	2.32	2.9	7.38	1.81	9.90	0.16	4.08	9.18	98.421	3.00
**10**	180	0.908	6.5	0.23	2.04	0.108	1.8	4.69	2.49	9.90	0.31	12.10	9.18	99.381	2.73

**Table A4:** Operating conditions estimated THREE parameters from best fitting and modeling errors used by AYDI A.

Experiment	*P* (bar)	$Q_{{\mathrm{CO}}_{2}}\;(\mathrm{kg}\;h^{-1})$	*d_P_*.10^4^ (m)	G (dimensionless)	*k_f_a*_0_ 10^2^ (s^−1^)	*k_s_a*_0_ 10^5^ (s^−1^)	*y_s_* 10^4^ (kg kg^−1^)	*k_f_* 10^6^ (m s^−1^)	*k_s_* 10^9^ (m s^−1^)	*D_AB_* 10^9^ (m^2^ s^−1^)	Sh	Re	Sc	r^2^	RMSE 10^2^
**1**	220	0.604	6.5	0.37	73.6	7.35	4.1	1.70	0.169	99.1	0.11	7.33	0.96	98.472	3.74
**2**	220	0.602	2.2	0.61	1.72	7.01	5.2	1.34	5.47	22.9	0.09	2.47	4.16	99.355	2.40
**3**	80	1.202	6.5	0.40	8.70	0.182	0.9	0.201	0.419	1440	0.14	53.53	0.06	99.718	1.73
**4**	80	1.209	6.5	0.37	8.95	0.107	0.9	0.206	0.246	1470	0.13	53.83	0.05	99.626	1.98
**5**	80	0.602	2.2	0.52	3.42	9.30	2.2	2.67	7.25	65.00	0.13	9.08	1.24	99.357	2.5
**6**	80	0.603	6.5	0.45	4.03	7.58	2.6	9.29	0.175	597.0	0.12	26.84	0.13	98.322	3.75
**7**	140	1.204	6.5	0.37	1.26	7.71	1.5	2.90	0.178	215.0	0.14	18.37	0.40	98.663	3.69
**8**	180	0.913	2.2	0.66	77.3	2.31	2.4	6.03	1.81	15.60	0.14	4.12	5.84	99.137	2.38
**9**	180	0.904	2.2	0.59	92.9	1.91	2.9	7.25	1.49	17.8	0.14	4.08	5.11	99.019	3.26
**10**	180	0.908	6.5	0.23	2.64	0.108	1.8	6.10	0.25	318.0	0.10	12.10	0.29	99.381	2.73
